# Severe trauma in Germany and Israel: are we speaking the same language? A trauma registry comparison

**DOI:** 10.3389/fpubh.2023.1136159

**Published:** 2023-05-02

**Authors:** Arielle Kaim, Moran Bodas, Dan Bieler, Irina Radomislensky, Gerrit Matthes, Adi Givon, Heiko Trentzsch, Israel Trauma Group, Christian Waydhas, Rolf Lefering

**Affiliations:** ^1^Israel National Center for Trauma and Emergency Medicine Research, The Gertner Institute for Epidemiology and Health Policy Research, Sheba Medical Center, Ramat-Gan, Israel; ^2^Department of Emergency and Disaster Management, Faculty of Medicine, School of Public Health, Sackler Tel Aviv University, Tel Aviv, Israel; ^3^Department of Trauma Surgery and Orthopedics, University Düsseldorf, Düsseldorf, Germany; ^4^Department for Trauma Surgery and Orthopedics, Reconstructive Surgery, Hand Surgery, Burn Medicine, German Armed Forces Central Hospital, Koblenz, Germany; ^5^Department of Trauma and Reconstructive Surgery, Hospital Ernst-von-Bergmann, Potsdam, Germany; ^6^Institut für Notfallmedizin und Medizinmanagement (INM), Klinikum der Universität München, LMU München, Munich, Germany; ^7^Department of Surgery, BG University Hospital Bergmannsheil, Bochum, Germany; ^8^Medical Faculty of University Duisburg-Essen, Essen, Germany; ^9^Institute for Research in Operative Medicine, University Witten/Herdecke, Cologne, Germany

**Keywords:** trauma registry system, Germany, Israel, trauma care quality, trauma, trauma registry comparison

## Abstract

**Background:**

Trauma registries are a crucial component of trauma systems, as they could be utilized to perform a benchmarking of quality of care and enable research in a critical but important area of health care. The aim of this study is to compare the performance of two national trauma systems: Germany (TraumaRegister DGU®, TR-DGU) and Israel (Israeli National Trauma Registry, INTR).

**Methods:**

The present study was a retrospective analysis of data from the described above trauma registries in Israel and Germany. Adult patients from both registries treated during 2015–2019 with an Injury Severity Score (ISS) ≥ 16 points were included. Patient demographics, type, distribution, mechanism, and severity of injury, treatment delivered and length of stay (LOS) in the ICU and in the hospital were included in the analysis.

**Results:**

Data were available from 12,585 Israeli patients and 55,660 German patients. Age and sex distribution were comparable, and road traffic collisions were the most prevalent cause of injuries. The ISS of German patients was higher (ISS 24 vs. 20), more patients were treated on an intensive care unit (92 vs. 32%), and mortality was higher (19.4 vs. 9.5%) as well.

**Conclusion:**

Despite similar inclusion criteria (ISS ≥ 16), remarkable differences between the two national datasets were observed. Most probably, this was caused by different recruitment strategies of both registries, like trauma team activation and need for intensive care in TR-DGU. More detailed analyses are needed to uncover similarities and differences of both trauma systems.

## Introduction

Worldwide, trauma or injury is a major health concern and one of the leading causes of death and disability (1). The distribution of trauma on the global, national and local levels differs. Correspondingly, heterogeneity exists in terms of its underlying causes, types of injury, and severity (2). A key component of a viable trauma system is a national program for tracking trauma patients, which comprises of a Trauma Registry (TR). Trauma registries or comprehensive data repositories regarding injured patients have made important contributions to improving trauma care throughout the past several decades (3–7). The great implication of trauma registries is in their potential to perform comparison of quality control and benchmarking at varying levels of analysis, which may have health policy implications (4, 5). Moreover, national programs for registering trauma patients have the capacity to support important research that is crucial for improving clinical practice and ultimately for saving lives.

Previously, comparative studies of data from trauma registries conducted on the international level have elicited noteworthy insights, which, for example, indicated the need for healthcare reforms through redistribution of resources (5, 8–10). Findings of Roudsari et al. (10) from a multi-center study suggest that pre-hospital care systems that dispatch a physician to the scene may be associated with lower early trauma fatality rates, but not significantly better outcomes regarding additional clinical measures (10). A study comparing the Navarra Major Trauma Registry of Spain and the Atlantic Pyrenees (France) conducted by de Segura et al. (8) indicated that despite allocation of greater resources and a more assertive approach, the French registry did not show better survival rates than the injured patients of Navarra (8). Additional comparisons of trauma registries conducted more recently between Germany and Hong Kong indicated significant differences in mechanism and distribution of major injuries, rates of surgical interventions/Intensive Care Unit (ICU) admissions, and mortality outcomes (9).

Both, Germany, and Israel have established a national program for registering trauma patients and monitoring the epidemiology of trauma. These programs rely primarily on a trauma registry, which covers a large percentage of patients with relevant injuries. These registries offer the unique opportunity to compare several aspects of trauma epidemiology and trauma care between both countries, as has been previously done. To date, no comparative study has yet been undertaken with respect to the performance of the trauma systems and patient outcomes concerning Israel and Germany. The objective of the current study is to compare performance of the respective systems incorporating the assessment of outcomes of adult (18+) major trauma victims over a 5-year period of 2015–2019 grounded upon data retrieved from the corresponding registries. As part of this comparison, the organizational framework of emergency care will be described.

## Methods

### Setting and registries

#### TraumaRegister DGU^®^

The TraumaRegister DGU® (TR-DGU) of the German Trauma Society (Deutsche Gesellschaft für Unfallchirurgie e.V., DGU) was founded in 1993 (11). The aim of this multi-center database is a pseudonymized and standardized documentation of severely injured patients.

Data are collected prospectively in four consecutive time phases from the site of the accident until discharge from hospital: (A) Pre-hospital phase, (B) Emergency room and initial surgery, (C) Intensive care unit, and (D) Discharge. The documentation includes detailed information on demographics, injury pattern, comorbidities, pre- and in-hospital management, course on the intensive care unit (ICU), relevant laboratory findings including data on transfusion, and outcome of each individual. The inclusion criterion is admission to hospital via the emergency room (trauma team activation) with subsequent intensive or intermediate care. Patients who reached the hospital with vital signs but died before admission to ICU were included as well.

The infrastructure for documentation, data management, and data analysis is provided by AUC—Academy for Trauma Surgery (AUC—Akademie der Unfallchirurgie GmbH), a company affiliated to the German Trauma Society. The scientific leadership is provided by the Committee on Emergency Medicine, Intensive Care and Trauma Management (Sektion NIS) of the German Trauma Society (DGU). The participating hospitals submit their data pseudonymized into a central database via a web-based application. Scientific data analysis is approved according to a peer review procedure laid down in the publication guideline of TR-DGU.

The participating hospitals are primarily located in Germany (90%), but a rising number of hospitals of other countries contribute data as well (at the moment from Austria, Belgium, Finland, Luxembourg, Slovenia, Switzerland, The Netherlands, and the United Arab Emirates). Currently, approx. 33,000 cases from over 650 hospitals are entered into the database per year. Participation in TR-DGU is voluntary. For hospitals associated with TraumaNetzwerk DGU®, however, the entry of at least a basic data set is obligatory for reasons of quality assurance.

This study was conducted according to the publication guideline of the TR-DGU and registered as project number 2021-005.

#### Israeli national trauma registry

The Israeli National Trauma Registry (INTR) was established in 1995, with the aim of providing a tool for improving the quality of care and treatment provided to trauma victims, to monitor injuries through epidemiological assessments, to assist in the creation of prevention programs, and to support the shaping of national health policy with respect to trauma care in Israel (6). The ultimate goal of the national program for registering trauma patients in Israel, in which the INTR is operating, is to save lives, reduce injuries, and prevent disabilities.

The data are collected at the hospital by the trauma registrars, monitored by the trauma coordinator, and is the responsibility of the trauma unit director. The data are entered into a computerized system and transmitted to the central database managed by the Israel National Center for Trauma and Emergency Medicine Research at the Gertner Institute for Epidemiology and Health Policy Research (Sheba Medical Center). The trauma unit and registrars at the hospital are responsible for the quality and accuracy of the data. However, after the data are received from the hospital and entered into the central database, logical and other checks are performed to ensure its quality and completeness. Missing, unclear or erroneous data is corrected and completed at the request of the National Center for Trauma and Emergency Medicine Research.

Participating in the national program for registering trauma patients are 21 trauma centers in Israel, including all six Level I trauma centers. All patients hospitalized after being admitted to the participating trauma centers’ emergency department (ED) due to injury and assigned an ICD-9-CM diagnosis code between 800 and 959.9 are included in the INTR. Those who died in the ED or were transferred to another hospital are also included. Not included in the registry are casualties who died prior to arriving to the hospital, patients who were not admitted to hospitals, and those discharged from the ED (not hospitalized).

The anonymized information collected on each of the patients in the registry includes close to 300 variables: demographic data, circumstances of the injury, type and severity of the injury, treatment at the scene, how patient arrived at the hospital, hospital departments for admission, diagnostic and surgical procedures, trauma resuscitation unit, ICU, length of hospitalization, destination upon discharge, outcome (discharge), and more. All injuries are coded according to the Abbreviated Injury Scale (AIS) by local trauma registrars.

Description of both registries and the settings are provided in Table 1.

**Table 1:** Description of organization of emergency care in Germany and Israel.

	Israel	Germany
Inhabitants	9.2 million	83.020 million
Area	22,145 km^2^	357,386 km^2^
Population density	415/km^2^	233/km^2^
Hospitals	26 hospitals (21 participate in INTR, where 95% of severe trauma cases are treated throughout the country)	750 participating hospitals (>95% of severe trauma cases covered)
Level of care	The hospital’s trauma center level is accredited by a governmental committee assigned by the Ministry of Health, according to designated protocols	Nearly all hospitals are organized in regional networks and certified as trauma center (TC) every 3 years by DGU
	Level 1 Hospitals: 6; treating 61% of all trauma patients	Level 1 (supra-regional TC): 114 hospitals; treating 65% of all trauma patients; on avg. 162 cases/year
	Level 2 Centers: 15; treating 39% of all trauma patients	Level 2 (regional TC): 228 hospitals; treating 29% of all trauma cases; on avg. 54 cases/year
		Level 3 (local TC): 347 hospitals; treating 6% of all trauma patients; on avg. 19 cases/year
Trauma registry	Founded in 1995.	Founded in 1993
	40,000 new cases/year	35.000 new cases/year
	Annual incidence rate: 27.35 per 100,000 inhabitants	Annual incidence rate: 16.86 per 100,000
	Total: 750,000 cases	Total: 400,000 cases
	Registry inclusion criteria: All patients hospitalized after being admitted to the participating trauma centers’ emergency department (ED) due to injury and assigned an ICD-9-CM diagnosis code between 800 and 959.9 are included in the INTR. Those who died in the ED or were transferred to another hospital are also included. Not included in the registry are casualties who died prior to arriving to the hospital, patients who were not admitted to hospitals, and those discharged from the ED (not hospitalized)	Open to other countries.
		Registry inclusion criteria: Admission to hospital via the emergency room (trauma team activation) with subsequent intensive or intermediate care. Patients who reached the hospital with vital signs but died before admission to ICU were included as well
Pre-hospital care	The prehospital level is primarily maintained by one of the emergency medical services (EMS)—Magan David Adom (a member of the International Federation of Red Cross)	Numerous organizations and providers throughout the country including professional fire departments and a network of 89 emergency helicopters
	There are currently approximately 1,200 salaried Emergency Medicine Technicians (EMTs) and 650 paramedics, there is a robust multi-level system of over 40,000 volunteers out of a total population of 9.2 million	Rendezvous system with ambulance car (paramedic-staffed) and emergency physician rapid response car or helicopter. Nearly all severe trauma cases are seen by an emergency physician on scene
	MDA has also three helicopters designated for quick evacuation of the sick and wounded from the periphery to central hospitals. Additionally, pre-hospital care on occasion is provided by the Israeli Defense Force Medical Corps (IDF-MC). This assistance begins in prehospital settings and typically culminates in Israeli civilian hospitals.	The destination of a patient is decided by the treating emergency physician together with the dispatch center
	Israel has a purely paramedic-based rescue system.	
Hospital emergency department	Patients with trauma are triaged to trauma centers. There, a dedicated trauma team attends to these patients. The trauma centers are affiliated to a surgical division and are staffed by a multidisciplinary team from various departments of a hospital	Multidisciplinary trauma team composed of anesthesiologists, trauma surgeons, radiologists, nursing staff and radiological technicians. Composition may vary according to trauma level center.
		Trauma team activation is an inclusion criteria for TR-DGU
Further resources	The National Trauma Council was appointed to advise the Ministry of Health on issues related to trauma.	Whitebook of trauma care, edited and regularly updated by DGU (12).
		National evidence-based guideline for polytrauma care (13).

### Study design and methodology

The present study was a retrospective analysis of data from the described above trauma registries in Israel and Germany. Data of a 5-year-period from January 2015 to December 2019 were extracted from the trauma registries (TR-DGU and INTR). The present study focuses on severe trauma patients, only adults (18 years old and plus) with ISS ≥16 were eligible. From the TR-DGU, cases outside of Germany were excluded. Furthermore, those suffering from drowning, poisoning, and hanging are excluded. Patients who died prior to or at arrival to the emergency department, transfer-in cases and those who were transferred (in or out) are excluded. Table 2 summarizes the inclusion and exclusion criteria.

**Table 2:** Inclusion and exclusion criteria for this comparative analysis.

Inclusion criteria	Exclusion criteria
Injury severity score ≥ 16	Severe burns (AIS 4+)
Age ≥ 18 years	Hanging
Time of admission: January 1, 2015 to December 31, 2019, inclusive	Drowning
	Poisoning
	Dead upon arrival at emergency department
	Transfers in from other hospital
	Transfer out within 48 h

Patient characteristics including age and gender were collected. Type, distribution, mechanism, and severity of injury, treatment delivered and length of stay (LOS) in the ICU and in the hospital were included in the analysis. High Falls were defined as falls from 3 m or above, 0–3 m (non-inclusive) as low falls, and 0 m as fall from the same plane. The discharge plan was also recorded as a secondary outcome. The mortality in both registries is documented as in-hospital mortality.

### Ethical standards

The protocol of this study has been approved by the Sheba Medical Center Ethics Committee (Approval number SMC-18-5138), as well as has received a waiver from ethic committee review by the University Witten/ Herdecke (number 64/2018). The present study is in line with the guideline for publication of the TR-DGU (registered as project number 2021-005), as well as the INTR. The research conducted is in line with the Declaration of Helsinki.

### Analysis

Descriptive analysis was used to explore similarities and differences between the registries. Frequencies are presented with number of cases and percentage. Continuous measurements were presented as mean with standard deviation (SD), or as median with inter-quartile range (IQR), based on the distribution. Formal statistical significance testing was avoided due to the large sample size. The detectable difference would be about 1.0% depending on the prevalence (alpha 0.05, power 0.80). Data from both countries were not merged but analyzed independently. German data were analyzed with SPSS statistical software package (version 26, IBM Inc., Armonk, NY, United States). Israeli data was analyzed with SAS V9.4 statistical software.

### Outcome measures

The primary outcomes of the study were the differences in outcomes such as in-hospital mortality, length of stay in hospital, and length of stay in ICU. Differences between patient characteristics, injury mechanism and patterns, and in-hospital management were secondary outcomes of the study.

## Results

Of approximately 500,000 total victims documented by the INTR, data of 12,585 trauma victims (from 21 trauma centers) admitted between 2015 and 2019 were extracted from the registry, according to the inclusion/exclusion criteria. Similarly, of a total number of 400,000 cases documented in the TR-DGU, 55,660 datasets (from 663 hospitals) were retrieved for this analysis. The number of datasets extracted from the TR-DGU was nearly five times that of the INTR. Furthermore, given the data provided in Table 3, the average number of severe trauma patients per hospital differs (over the 5-year period), with 599 severe trauma patients per hospital (12,585 patients /21 hospitals) documented in Israel, while correspondingly only approximately 84 (55,660/ 663) in the context of Germany,

**Table 3:** Characteristics of adult severe trauma patients in Israel and Germany.

	Israel *n* = 12,585	Germany *n* = 55,660
**Patient characteristics**		
Age (years), mean (SD)	54.0 (23.4)	55.3 (20.5)
Age ≥ 60 years, *n* (%)	5,543 (44.0%)	25,864 (46.5%)
Males, *n* (%)	8,958 (71.2%)	38,855 (69.8%)
**Injury mechanism**		
Penetrating trauma, *n* (%)^a^	927 (7.4%)	1,933 (3.6%)
Suspected suicide, *n* (%)^a^	235 (1.9%)	3,147 (5.8%)
Suspected violence, *n* (%)^a^	962 (7.6%)	1,214 (2.2%)
Road traffic accidents, *n* (%)	5,170 (41.1%)	27,117 (49.3%)
Car, *n* (%)	2,190 (17.4%)	11,108 (20.2%)
Motor bike	962 (7.7%)	6,465 (11.8%)
Bicycle	416 (3.3%)	5,189 (9.4%)
Pedestrian	1,177 (9.4%)	3,436 (6.2%)
Stabbing or piercing, *n* (%)	360 (2.9%)	864 (1.6%)
Gunshot, *n* (%)	322 (2.6%)	390 (0.7%)
Falls, *n* (%)	5,631 (44.7%)	23,755 (43.2%)
High fall (3+ m)	876 (7.0%)	9,347 (17.0%)
Low falls (>0/<3 m)	1,260 (10.0%)	14,408 (26.2%)
Ground level fall (0 m)	2,920 (23.2%)	(contained in low falls)
Unknown height	575 (4.6%)	---
**Injury pattern (ISS body regions)**		
Head (AIS 3+), *n* (%)	7,427 (59.0%)	28,845 (51.8%)
Thorax (AIS 3+), *n* (%)	5,786 (46.0%)	30,951 (55.6%)
Abdomen (AIS 3+), *n* (%)	2,407 (19.1%)	7,989 (14.4%)
Extremities (AIS 3+), *n* (%)	2,486 (19.8%)	16,040 (28.8%)
Injury severity score (ISS), median (IQR)	20 (17–26)	24 (18–29)
ISS 16–24, *n* (%)	7,834 (62.2%)	29,031 (52.2%)
ISS 25+, *n* (%)	4,751 (37.8%)	26,629 (47.8%)
**Admission status**		
Transport to hospital, *n* (%)		
Helicopter	503 (4.0%)	12,972 (24.2%)
Ground transport by EMS	n/a	39,950 (74,5%)
Private	n/a	660 (1.2%)
Admitted during night time (6 pm–6 am), *n* (%)	4,853 (38.6%)	20,706 (37.2%)
Syst. BP < 90 mmHg in ED, *n* (%)	726 (5.8%)	1,304 (2.5%)
Unconsciousness (GCS ≤ 8) *n* (%)	1,811 (14.4%)	13,884 (24.9%)
**Hospital care**		
Intubation in ED, *n* (%)	1,660 (13.2%)	2,969 (10.5%)^#^
Fluid administration in ED, *n* (%)	4,701 (37.4%)	21,493 (91.5%)^#^
CPR performed in ED, *n* (%)	179 (1.4%)	1,171 (4.1%)^#^
Sonography (abdomen; FAST) in ED, *n* (%)	3,934 (24.1%)	48,447 (86.9%)		Israel *n* = 12,585	Germany *n* = 55,660
X-ray of thorax in ED, *n* (%)	3,459 (64.3%)	16,569 (30.0%)
Any CT (cranial, selected organs, whole-body) in ED, *n* (%)	11,395 (90.5%)	52,151 (93.7%)
Blood transfusion (before ICU), *n* (%)	1,581 (12.6%)	6,446 (11.7%)
**Intensive care**		
Treated in the ICU, *n* (%)	4,806 (38.2%)	51,227 (92.0%)
Intubation/ventilation, *n* (%)	2,683 (44.2%)	27.251 (53.2%)
Mortality in patients treated on ICU, *n* (%)	658 (13.7%)	8,904 (17.4%)
Length of stay on ICU (days), Median (IQR)	4 (2–13)	4 (1–11)
**Outcome**		
Died among ISS 25+, *n* (%)	996 (21.0%)	8,832 (33.2%)
Died in the emergency room, *n* (%)	181 (1.4%)	1,572 (2.8%)
Died within 24 h, *n* (%)	410 (3.3%)	5,789 (10.4%)
Died in hospital, *n* (%)	1,199 (9.5%)	10,818 (19.4%)
Hospital length of stay (days), Median (IQR)	6 (3–14)	13 (7–23)
Discharge (survivor only), *n* (%)		
Home	7,988 (70.2%)	25,042 (58.8%)
Rehabilitation	2,801 (24.6%)	12,295 (27.4%)
Transfer to other hospital	—	5,702 (12.7%)
Other destination	597 (4.7%)	1,803 (4.0%)

Mean is presented with standard deviation, median with Interquartile Range (IQR).

^#^Data are available only from hospitals using the standard documentation form of TR-DGU.

a Categories may overlap regarding context of injury (e.g., intentional etc.) and mechanism (e.g., stabbing).

The patient sample characteristics are shown in Table 3. The sex and age distribution are similar in both countries. Males in Israel accounted for 71.2% of the dataset, while 69.8% in the TR-DGU. The mean age in Israel was 54.0 (SD 23.4), while in Germany the average age was 55.3 (SD 20.5).

Regarding injury mechanism and injury profile, in Israel the most common mechanism of injury among all injuries was fall at ground level (23.2%), followed by road traffic collisions with a car (17.4%). In Germany, low falls (including ground level falls) were the most common mechanism of injury (26.2%) followed by road traffic collisions with a car (20.2%). Penetrating injuries were more common in Israel (7.4%) as compared to Germany (3.6%). Furthermore, compared to Israel, there were more suspected suicides in Germany (5.8 vs. 1.9%). Contrastingly, in Israel suspected violence was a greater source of injury as compared to Germany (7.6 vs. 2.2%), with a greater number of stabbing (2.9 vs. 1.6%) and gunshot injuries (2.6 vs. 0.7%, see Table 3).

Patients in Germany sustained higher injury severity as reflected by the Injury Severity Score (ISS) with medians of 24 [IQR 18–29] compared to 20 [IQR 17–26] in Israel. Also, patients with critical injuries, defined as ISS 25+, were more prevalent in Germany (48%) than in Israel (38%). Distribution pattern of injuries also varied. While serious head injury (AIS 3+) was observed more frequently in Israel (59 vs. 52%), German patients suffered from more thoracic trauma and injuries to the extremities (Table 3).

As the INTR has limited access to pre-hospital data, the focus of comparison was placed on admission status and in-hospital patient management. In Germany, 90% of severely injured patients were directly brought into the hospital where their definite treatment was performed. Only 10% were transfers from other hospitals. 74% of these transfers were performed within the first 6 h after primary admission. In Israel, approximately 84% of patients were brought directly into the hospital where they received medical care. Of the 16% transferred, 76% of these transfers were performed in the first 6 h after primary admission.

The in-hospital care revealed some differences in procedures and diagnostics. Sonographies were performed much more frequently in Germany (86.9 vs. 24.1%) while the rate of CT scans was rather similar (93.7 vs. 90.5%). In Israel, *x*-ray evaluation of the thorax was performed more than twice as frequent as in Germany (64.3 vs. 30.0%) but blood transfusion rate was similar (12.6 vs. 11.7%). Fluids were administered in the context of Germany more than twice as much as in the context of Israel (91.5 vs. 37.4%).

Requirement of intensive care substantially differed as well. In Israel, only 38.2% of victims were admitted to an ICU while in Germany, 92.0% received intensive care. For those treated in the ICU, the median LOS in both countries was 4 days. Median hospital LOS in the acute care hospital for trauma victims was much shorter in Israel versus Germany, 6 and 13 days, respectively.

Table 3 also demonstrates the differences in outcomes and mortality between both settings. The data indicate lower in-hospital mortality in Israel (9.5 vs. 19.4%) among trauma victims during the study-period. Similarly, the data confirmed that observed mortality in the first 24 h was substantially less in Israel (3.3 vs. 10.4%). Mortality differences were also found among patients treated in the ICU, where 13.7% died in Israel vs. 17.4% in Germany. Concerning discharge of survivors, 70.2% were discharged home in Israel while in Germany, 58.8% were discharged home, and another 12.7% were transferred to another hospital for a few days of further treatment.

## Discussion

The present comparative study exposes interesting and noteworthy differences between the trauma systems in Israel and Germany, reflected by the results of both trauma registries, the INTR and the TR-DGU First, while in both registries, small to large hospitals are included, the TR-DGU registry includes a much higher number of hospitals, as compared to the 21 trauma centers which provide data for the INTR. Accordingly, the calculated average number of severe trauma patients treated per hospital are much higher in Israel. Regarding the demographic distribution, the study shows rather similar trauma populations. Injury mechanisms differ in terms of traffic collisions (more in Germany) and penetrating injuries (more in Israel).

Although only patients with ISS ≥16 points were included, in-hospital mortality rate was considerably lower in Israel than in Germany (9.5 vs. 19.4%) and median hospital LOS was shorter (6 vs. 13 days), respectively. What might be the reason for this? Since both countries have highly developed systems of acute trauma care and education, it does not seem plausible that this difference is a result of differences in quality of care. It is possible that the patient groups considered in this study are not fully comparable, despite intended comparability between the two datasets through the investigation of severe (ISS ≥16) trauma cases. A restriction to more severe cases (ISS 25+) still shows a difference in mortality (21.0 vs. 33.2%). There is an obvious difference in injury severity (on average four points higher in German patients), and also need for intensive care shows a much higher rate in Germany (92 vs. 38%). The low rate of admission to the ICU in INTR may partially be caused by the shortage of ICU beds in Israel as described by Zisk-Rony et al. (14). On the other hand, besides trauma team activation, potential need of critical care is an inclusion criterion for TR-DGU, and ICU-treatment may increase the risk of adverse outcomes. Unfortunately, there is no identical prognostic score available in both registries, which could help to explain the different mortality rates. On the pre-hospital level, the substantial differences in traveling distance to hospitals and geographic areas between the two countries (Table 1) may also partially explain the outcomes. Furthermore, there are significant differences in the pre-hospital strategies, with a paramedic-based system in Israel (operating under the Anglo-American model), and a physician-based system in Germany where almost all severely injured trauma case receive care from an emergency physician already at the scene (5, 9, 14, 15). A paramedic-based system usually prioritizes hospital transport and minimization of pre-hospital time and thus has shorter on-scene times (“scoop and run” system) (14–18), while contrastingly, the physician-based system may result in longer pre-hospital times, as this approach more closely resembles the “stay-and- play” approach of treating trauma casualties on the scene and aims to transport patients directly to dedicated trauma centers, while bypassing smaller hospitals (19–22). Despite the findings of this study, Knapp et al. (23) in a meta analysis, has indicated that prehospital management of severely injured patients by EMS teams which include a physician seems to be associated with lower mortality (with non-significant trends when excluding the confounder of helicopter transport) (23). The controversy between the two strategies to date is not yet conclusive and requires further examination with additional trauma registries with more uniform inclusion/ exclusion criteria, with different pre-hospital strategies. An example of a future study, which may bring about more conclusive findings, may be to compare the INTR with the Dutch National Trauma Registry (DNTR), which have grossly the same inclusion criteria with different prehospital strategies (24). Similarly, a similar comparison should be conducted with the TR-DGU.

At the hospital level, previous literature has pointed to the fact that the greater the number of severely injured patients the hospital treats and the greater the centralization of patients, the better the survival and outcomes (25). This may be applicable in the context of our current findings where on average the number of patients treated in Israel is higher. Hietbrink et al. demonstrated that following the organizational changes in the Netherlands, centralization of patients allowed for consolidation of experience and knowledge, resulting in overall major improvements in efficiency with lower length of stay and mortality reduction (25).

Probably there is a substantial group of patients with ISS 16+ who were admitted via the shock room, however were not requiring intensive care, which was missed by TR-DGU. These patients seem to have a better prognosis. For example, a seriously injured patient with ISS 16+ who was admitted via the shock room, however, was not admitted to the ICU, would not be included in TR-DGU. In comparison, the INTR includes all patients with a specific trauma ICD, irrespective of the type of admission, thus such a patient would be included. This system of patient acquisition would be more complete than the German system, which tends to have concentrated on the more severe cases.

In order to analyze the mortality differences in more detail, some prognostic estimates would be helpful. The well-known but outdated TRISS method would be one option, or the prognostic system of the Revised Injury Severity Classification, version II (RISC II) as is applied in the TR-DGU (26). Future studies will be needed to analyze these differences in more detail.

Regarding mechanism and pattern of injuries, variability is shown. The higher frequency of injuries in Israel to the head is related to the high frequency of low falls and ground level falls (27, 28). In Germany, the higher percentage of injuries to the thorax and the extremities was observed which could be explained by more traffic collisions (29). In Israel, the number of suspected suicides was lower, while penetrating injuries were twice as frequent in Israel, which may be explained given the Israeli context in which terror-related injury is more frequent according to the Global Terrorism Index (30–32). These observations may reflect the need for certain services within the trauma system to be further expanded, for example neurosurgical services in the context of Israel. This would provide for the appropriate availability of resources given the injury patterns described above and the respective needs.

The data on long-term outcomes are still lacking as no-follow up is conducted with patients once they are discharged from both registries. Future studies may benefit from establishing a follow-up system in both trauma registries to better reveal long-term outcomes of trauma victims for an improved benchmark of the quality of trauma care provided. Furthermore, similar studies of this nature must be continued to be implemented on regional, national, and international levels in order to better reveal best practices in the field. It is also desired to agree on uniform inclusion criteria for trauma registries, which would facilitate international comparisons enormously and application of standardized prognostic instruments such as RISC II.

### Limitations

A primary limitation of this study is the different system criteria for ICU admission in the INTR and TR-DGU, which may significantly impact on the comparability of these two complex data sets and the study findings. In addition to this, long-term performance metrics of trauma victims are unavailable in both countries, which could provide an improved benchmark of the quality of trauma care provided.

### Conclusion

The present study evaluates and compares the performance of two different, but comparable national programs for registering trauma patients in central Europe and the Middle East centered upon data retrieved of severely injured victims in the respective trauma registries. Several differences are found between the trauma systems and the outcomes of victims, which most probably are based on different inclusion criteria, for example resulting from need of trauma team activation and intensive care in TR-DGU. In the context of the current study, “severe trauma” in both registries does not seem to have synonymous meaning, despite intended comparability resulting from the nature of ISS 16+ patient’s examination. The primary outcomes of this study provide the capacity to account for differences between the German and Israeli trauma systems in a manner that may highlight crucial and global aspects of trauma care. This study is added to a growing body of literature that explores differences between trauma registries. We call for additional studies to facilitate a deeper understanding of trauma care differences around the globe. Future studies should aim to ensure better uniformity in inclusion and exclusion parameters to ensure improved comparability, as well as include severity adjustment based on prognostic estimates and evaluate long-term performance metrics of trauma victims.

## Israel trauma group

A. Acker, N. Aviran, H. Bahouth, A. Bar, A. Becker, A. Braslavsky, D. Fadeev, A. L. Goldstein, I. Grevtsev, I. Jeroukhimov, A. Kedar, A. Korin, B. Levit, A. D. Schwarz, W. Shomar, D. Soffer, I. Schrier, M. Venturero, M. Weiss, O. Yaslowitz, and I. Zoarets.

## Data availability statement

The raw data supporting the conclusions of this article will be made available by the authors, without undue reservation.

## Ethics statement

The studies involving human participants were reviewed and approved by Sheba Medical Center Ethics Committee. Written informed consent for participation was not required for this study in accordance with the national legislation and the institutional requirements.

## Author contributions

MB and RL conceived the study. AK, DB, IR, GM, AG, HT, ITG, and CW supervised the conduct of the trial and data collection and undertook recruitment of participating centers and patients and managed the data, including quality control. AK, IR, AG, and RL provided statistical advice on study design and analyzed the data. AG, IR, and RL chaired the data oversight committee. AK drafted the manuscript and takes responsibility for the paper as a whole. All authors contributed to the article and approved the submitted version.

## Conflict of interest

The authors declare that the research was conducted in the absence of any commercial or financial relationships that could be construed as a potential conflict of interest.

The handling editor VB declared a shared affiliation with the author HT at the time of review.

## Publisher’s note

All claims expressed in this article are solely those of the authors and do not necessarily represent those of their affiliated organizations, or those of the publisher, the editors and the reviewers. Any product that may be evaluated in this article, or claim that may be made by its manufacturer, is not guaranteed or endorsed by the publisher.
